# Dictionary of Practical Medicine, Comprising General Pathology, the Nature and Treatment of Diseases, &c.

**Published:** 1845-11-01

**Authors:** 


*Dictionary of practical Medicine, comprising general Pathology, the nature and treatment of diseases, &c.—by James Copland, M. D.—Edited with additions by C. A. Lee, M. D.*

We are fortunate in living in an epoch of Cyclopœdias and Medical Dictionaries! This remark may at first seem to possess little significancy, yet upon reflection it will be found to contain an important and forcible truth. All who are qualified to judge of the matter by observation or experience, must be aware of the many, great and peculiar difficulties in the way of uniting with the active duties of our profession, those facilities, and habits of study which are necessary to constitute a well informed and truly scientific practitioner. Prominent among these difficulties are the great extent and variety of the subjects involved, together with the nature of medical duties, which occur without forewarning, are immediate in their requisitions, and defy all systematic arrrngement. To these we are obliged to add the *res angusta*, which constitute the rule, rather than the exception with the profession of this country. Few members of the profession are able to accumulate libraries, or to supply themselves with the majority of the medical works which are issued from the press. This may be a mortifying acknowledgment, but there is, in reality, less reason for humiliation than if it were the *will*, rather than *poverty* which refuses consent. Who can blame the decision, which, when families are to be cared for, or debts to be paid, sacrifices, it may be with a heavy heart, aspirations for improvement, because, if the latter be indulged, the former objects are unattainable! We might follow this train of reflection, but it is here merely incidental. The fact is alone important to our present purpose.

Under existing circumstances it is obvious, that works which bring together in a compact and accessible form the latest and best information on all the various subjects which medicine embraces, and which may be procured at a small expense, are calculated to remedy in a great degree some of the difficulties which have just been alluded to, and to fulfil a great desideratum for the medical practitioner. Two such works have within a few years been published in England. The Cyclopœdia of practical medicine which was noticed in our first number, and the work, the title of which is prefixed to this article. The latter is now in progress of publication in this country by the well known and enterprising publishing house of the Harpers' of New York. Possessing these works, the physician is able whenever any subject presents itself to his reflections, or is suggested by facts falling under his observation, to refer at once to them for a digest of all the information which our recent medical literature contains. And if he avail himself faithfully of this advantage, with these works alone he may acquire a vast amount of useful knowledge, and, in fact, become a learned and accomplished practitioner.

This plan of a multum in parvo, is precisely what is required by the practical members of our profession, and will do very much toward diffusing medical knowledge, and enhancing medical acquirement. If we may be allowed a comparison which the non-medical reader would say savored of the shop, these works bear to the general bibliography of the profession, somewhat the same relation as the medicinal extracts to the plants from which they are derived, containing all the essential principles wished for, in a concentrated, portable form, very convenient for practical use.

When it is considered that it can only happen after intervals of many years that works of this kind will be projected and executed by competent individuals; that in a short time those which now represent the condition of our science will be chiefly valuable as records of the errors and imperfections of the past—such is the rapid progress of knowledge—we have every reason to congratulate ourselves that their publication is contemporaneous with ourselves. We repeat the remark, therefore, with which we commenced, that 'we are fortunate in living in an epoch of Cyclopœdias and Medical Dictionaries!'

The Dictionary of Copland is nearly, if not quite as comprehensive in its plan, as the Cyclopœdia. It is justly styled by the London Gazette a miracle of industry, and we are happy to learn from a late number of the

Medico-chirurgical that the author, notwithstanding his almost incredible literary labors, is becoming daily more robust in his physical health!— This is adduced in the journal referred to, as evidence that intellectual labors do not necessarily injure the energies of the body.

We are not prepared to make any critical comparison of the two works. Each doubtless has its advantages, and it is desirable that both should be in the hands of the practical physician. Dr. Copland's work comprises a copious bibliography, which was omitted (rather injudiciously we think) in the American edition of the Cyclopœdia. Dr. Lee, the American Editor of the Dictionary, has earned the confidence of the profession, and will doubtless enhance the value of the work for the American reader.— We are gratified to learn from a late number of the New York Journal that the success thus far is satisfactory to the publishers. We would, in conclusion, hint to our brethren that a work which appears in successive numbers is purchased by monthly instalments without annoyance, when the aggregate amount disbursed at once, might, perhaps, be somewhat inconvenient. We hope none of our sensitive friends will take offence at this suggestion. We are free to say, haud inexpertus loquor.

